# Improving Glaucoma Diagnosis Assembling Deep Networks and Voting Schemes

**DOI:** 10.3390/diagnostics12061382

**Published:** 2022-06-02

**Authors:** Adrián Sánchez-Morales, Juan Morales-Sánchez, Oleksandr Kovalyk, Rafael Verdú-Monedero, José-Luis Sancho-Gómez

**Affiliations:** Departamento de Tecnologías de la Información y las Comunicaciones, Campus Muralla del Mar, Universidad Politécnica de Cartagena, 30202 Cartagena, Spain; adrian.sanchezm@edu.upct.es (A.S.-M.); juan.morales@upct.es (J.M.-S.); rafael.verdu@upct.es (R.V.-M.); josel.sancho@upct.es (J.-L.S.-G.)

**Keywords:** glaucoma, retinal images, deep learning, ensemble, soft voting

## Abstract

Glaucoma is a group of eye conditions that damage the optic nerve, the health of which is vital for good eyesight. This damage is often caused by higher-than-normal pressure in the eye. In the past few years, the applications of artificial intelligence and data science have increased rapidly in medicine especially in imaging applications. In particular, deep learning tools have been successfully applied obtaining, in some cases, results superior to those obtained by humans. In this article, we present a soft novel ensemble model based on the *K*-NN algorithm, that combines the probability of class membership obtained by several deep learning models. In this research, three models of different nature (CNN, CapsNets and Convolutional Autoencoders) have been selected searching for diversity. The latent space of these models are combined using the local information provided by the true sample labels and the *K*-NN algorithm is applied to determine the final decision. The results obtained on two different datasets of retinal images show that the proposed ensemble model improves the diagnosis capabilities for both the individual models and the state-of-the-art results.

## 1. Introduction

The automatic analysis of the image of the retina for the detection of diseases that cause blindness has been a subject of great scientific activity in recent years. In this article, we are interested in glaucoma, a progressive disease of the optic nerve caused by high intraocular pressure due to poor drainage of ocular fluid. Clinically, it translates into a progressive and irreversible loss of the visual field that progresses to total loss of vision. Currently, it is the second leading cause of blindness in the world [1] with the incidence of the disease increasing with age. This is observed statistically in that it affects one of each two hundred people under fifty years of age and one in ten over eighty years of age. Glaucoma is an initially asymptomatic disease, which is why it is generally detected in very advanced stages when the loss of the visual field is very manifest and irreversible. At the moment, there is no cure for established glaucomatous damage, and therefore early detection and prevention is the only way to avoid progression to total vision loss. The benefit of early diagnosis justifies carrying out screening campaigns. However, given the saturation of medical services at all levels, it would be desirable to automate these studies as much as possible and transfer them to primary care health centers in order to increase the number of the population analyzed without increasing costs.

**Figure 1:**
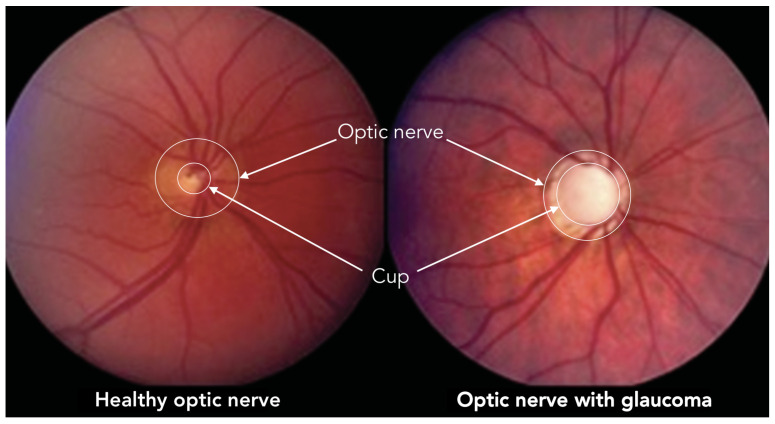
Optic nerve and cup in healthy and glaucoma optical fundus images.

According to scientific literature, the diagnosis of glaucoma is based mainly on three studies: the measure of intraocular pressure using tonometry, the examination of the visual field through campimetry, and the measurement of the relationship between the dimensions of the optic nerve excavation (Cup) and the optic nerve head (Optic Disc). This quantitative relation, known as Cup-to-Disk Ratio (CDR), is usually computed from fundus images [2] as a ratio of the diameters of both roughly circular shapes, but also it can be approximated as a ratio of both areas. In the case of glaucoma diagnosis by retinal image analysis, this last method is the most widely used. Figure 1 illustrates a couple of fundus images, one corresponding to a healthy eye (CDR = 0.3) and another with glaucoma (CDR = 0.9), where the optic disc and the excavation were also depicted.

Recently, the so-called Deep Learning (DL) has emerged with special force. DL can be considered as Machine Learning (ML) techniques that serve to model high-level abstractions in data using architectures composed of multiple non-linear layers forming a Deep Neural Network (DNN). In this way, better characteristics are obtained for later classification. In recent years, several works have been published [3,4,5,6] showing the success of DL in the field that is the subject of this work: the detection of glaucoma using deep network techniques. In all of them, Convolutional Neural Networks (CNN) are used in their deep version as they are universal approximators designed to work especially with images. However, these networks, although they present a clear advance with respect to conventional ML algorithms, present an intrinsic limitation since they are not capable of preserving spatial relationships between objects in the image, the spatial characteristics are lost, in such a way that an image that contains two eyes, a nose and a mouth will be detected by CNN as a face regardless of where these elements are located. To solve this problem, G. Hinton (one of the parents of DL and CNN) presented in November 2017 the Capsule Networks (CapsNets) [7]. These networks incorporate two very important novelties. On the one hand, network activations change, in such a way that they not only detect image characteristics (such as the presence of a mouth or an eye), but also detect their properties (size, orientation, position, etc.). These particular activations are called Capsules The second contribution is the way in which they are trained: by means of a dynamic routing algorithm that groups the capsules of a level to form capsules of higher levels (parent capsules), and calculates the outputs of the capsules.

Autoencoders are learning machines that produce outputs equal to inputs. Self-associative models of multilayer structures (Multilayer Perceptrons, MLPs) were studied to obtain reduced non-linear representations of the original data, obtaining good results with deep structures instead of single-layer networks [8]. Two types of deep AEs stand out: Stacked Denoising Autoencoders (SDAEs) [9] and Convolutional AEs (CAEs) [10]. The former are deep MLPs that add noise to entrances forcing clean entrances to rebuild during training. In addition, they allow layer-by-layer deep machine building, introducing non-linear effects by means of the use of expansive/contractive architectures. Convolutional Autoencoders (CAEs) are a variant of the previous ones that use convolutional instead of sigmoidal or ReLu layers. In this way, they are more appropriate for working with images since they can extract data-driven features directly from three-dimensional data (for example, RGB images).

Ensemble learning consists of the combination of different models to improve the generalization capabilities of the best individual machine, ref. [11]. For ensemble learning to be effective, there must be diversity in the different models. This diversity is translated into different partitions of the original space and can be obtained in different ways, for example, using different representations of the original training data and/or different learner designs, that is, different architectures, optimization algorithms and hyperparameters.

When building an ensemble, several aspects have to be taken into consideration. In particular, the following are of particular importance [12]: how diversity is introduced into the ensemble, the number of learners, the order in which these learners are trained (sequential or parallel) and, finally, the aggregation strategy to obtain the final prediction from the individual predictions. Respect to the last strategy, it can be (1) a linear combination, e.g., sum, median, maximum, minimum or weighted sum functions, (2) a voting strategy, e.g., majority voting, plurality voting, weighted voting or soft voting, (3) meta-combiner, which trains another learner, called meta-model, on the predictions of the base learners.

In this article, we propose a novel soft voting method that combines the probability of class membership obtained by three different machine learning models: CNN, CapsNet and CAE. This is an ensemble like method where the voting scheme output is provided by the largest summed probability from models.

This article is structured as follows. In the next section, a brief revision of the used deep learning models is presented. In Section 3, all aspects related to the experiments are described. In particular, it includes the description of the data sets, the design and training of the different models, as well as the results obtained and their discussion. The conclusions close the article.

## 2. Materials and Methods

According to previous studies, classical image processing methods are encouraging but they only focus on either geometrical properties or non-geometrical properties. Deep learning architectures are frequently a good option in image processing and have demonstrated to get good results in glaucoma detection. We will implement several deep architectures and evaluate their performance. In this section, we first briefly present these machines and then we explain how their outputs can be combined creating a novel ensemble method that improves the results obtained by each of the individual models.

### 2.1. Convolutional Neural Networks

Convolutional Neural Networks (CNN) are one of the engines that have driven the breakthrough of DL in recent years, being the preferred option for many computer vision tasks [13,14]. Inspired by the organization of the Visual Cortex, CNN are composed by individual neurons that respond to stimuli only in a restricted region of the visual field known as the Receptive Field. A collection of such fields overlap to cover the entire visual area.

**Figure 2:**
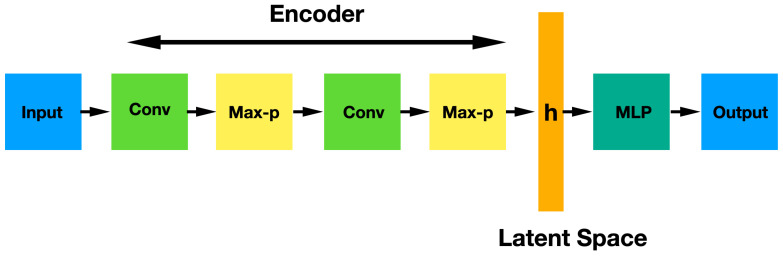
CNN architecture showing the sequential convolutional and max pooling layers and the final MLP classification after a flatten operation.

A CNN is a sequence of three different kinds of layers: convolutional layer (with non-linear ReLu activation), pooling layer and fully-connected layers. The convolutional and pooling layers are usually applied several times successively in order to achieve a deeper image representation by means of activation maps that contain the features which are critical for getting a good prediction. The resulting image can be flattened thus obtaining a vector **h** that is finally classified by a set of fully-connected layers. The vectorial space of vectors **h** is called latent space. Figure 2 shows the architecture of a CNN network.

The most relevant advantage of a CNN is the automatic feature extraction for the given task. Besides this, CNN have other important advantages mainly by using convolution and pooling operations. In particular, pooling

- makes the input representations smaller and more manageable;
- reduces the number of parameters and computations in the network, therefore, controlling overfitting;
- increases the field of view of higher layers thus allowing more general features of the input image to be obtained;
- makes the network invariant to small transformations, distortions and translations in the input image: a small distortion in the input will not change the output of pooling, since we take the maximum/average value in a local neighborhood.

Meanwhile convolution:

- helps us to obtain an object recognition method that is almost invariant under translation, which supposes a very powerful feature since we can detect objects in an image no matter where they are located.

Nevertheless, CNN present several limitations:

- CNN lack of ability to be invariant to large transformations, distortions and translations in the input image. Although CNN solves this problem slightly by using max pooling and convolution, these are simply a bad approach to the solution;
- max pooling loses valuable information;
- the internal representation of a CNN does not take into account the spatial relationships between objects, nor the existing hierarchy between simple objects and the composite objects of which they are a part.

### 2.2. CapsNets

There are many research proposals to address the limitations of CNN [15,16,17,18]. Among them, the so-called Capsule Networks (CapsNets) stand out. They are a novel structure of CNN which simulates the visual processing system of human brain and were proposed by G. E. Hinton to solve the intrinsic problems of CNN [7,19]. In particular, to achieve CNN with internal data representation that take into account spatial hierarchies between simple and complex objects.

**Figure 3:**
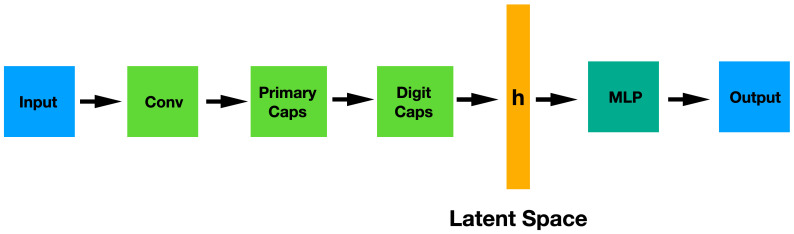
CapsNet architecture showing the convolutional section (encoder), the latent space and MLP classifier.

These networks are composed by capsules that, like the classical neurons, are non-linear computation nodes of a learning machine. Nevertheless, there is a remarkable difference between them: in a capsule, both the input components and the output are vectors instead of scalars, as it happens in a neuron. The length of the output vector denotes the probability that the entity (an object, for example) exists and the state of the detected entity is encoded in the direction of the vector. The architecture of this type of networks is represented in Figure 3. This architecture contains two non-linear capsule layers: a first convolutional layer, the so-called PrimaryCaps and then the DigitCaps. The flatten representation of the PrimaryCaps is the latent space **h** where an MLP network will try to solve the problem.

The most important characteristic of the CapsNets is that their output components, called “instantiation parameters”, are equivariant. This means that they allow maintaining the information of spatial relationships between the object components, which makes the network invariant to the viewpoint. Thus, a relevant consequence of this property emerges: a CapsNets can identify new, unseen variations of the class without ever being trained on them.

Equivariance is due, in part, to a new training algorithm called routing by agreement, which ensures that the output of a capsule is sent to an appropriate parent in the layer above. In particular, each output of the lower level capsules is multiplied by a coefficient and sent to every capsule in upper level layer. The routing algorithm increases or decreases these coupling coefficients according to the agreement between the output of the lower capsule and those of the layer above.

**Figure 4:**
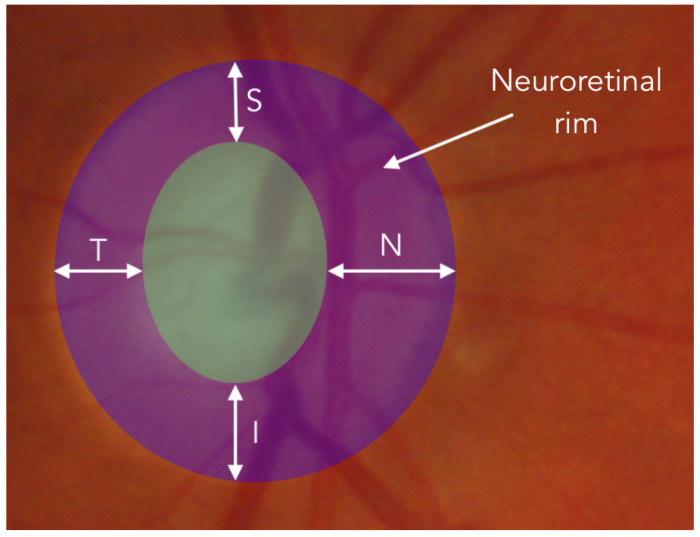
Representation over an ocular fundus image of the tikckness measurement of neuroretinal rim in the four directions: Inferior (I), Superior (S), Nasal (N) and Temporal (T).

The CapsNet has been chosen precisely to analyze whether the equivariance is a key point in order to detect the glaucoma in optical fundus images. For our problem, in particular, the CapsNet could help taking into account the ISNT rule. This rule, is used by ophthalmologists to measure the thickness of the neuroretinal rim, by estimating the thickness of the neuroretinal rim in four orthogonal directions: Inferior (I), Superior (S), Nasal (N) and Temporal (T). For healthy patients this rule usually satisfies the inequality I≥S≥N≥T. As shown in Figure 4, according to the measurement locations, accomplishing the ISNT rule also implies a spatial relationship between each above-mentioned thickness. Therefore, it is believed that CapsNets in this particular case could help to improve the results of common CNNs.

### 2.3. Convolutional Autoencoders

A conventional Autoencoder (AE) is generally made up of a first layer (encoder) that transforms the input features of the data to an intermediate latent space h, and a second layer (decoder) that, starting from h, tries to recover the values of the original input. Considering E(·) as the encoder function and D(·) that of the decoder, the objective of the training is to minimize the mean squared errors (MSE) between its input and output in all samples, that is,

(1) $$\min_{w_{e},w_{d}}\frac{1}{N}\sum_{j=1}^{N}{∥D_{w_{d}}\left(E_{w_{e}}\left(x_{j}\right)\right)-x_{j}∥}^{2}$$

where

(2) $$\begin{array}{cc} E_{w_{e}}\left(x\right) & =a(w_{e}^{T}x)\equiv h \end{array}$$

(3) $$\begin{array}{cc} D_{w_{d}}\left(h\right) & =a(w_{d}^{T}h) \end{array}$$

being *N* the number of training samples, *a* the activation function (ReLu or sigmoid), and x and h vectors.

To avoid the identity mapping, the **h** dimension use to be chosen lower than **x** dimension and **x** is perturbed by an additive noise obtaining the so-called Denoising AE (DAE), which can be stacked to build a deep network that provides multiple representation levels of the data (output of the hidden layers) where data can be easily classified. This is the Stacked DAE (SDAE) [9].

Here we focus on deep Convolutional Denoising Autoencoders (CDAE), as they are the right version to model and classify image data. To improve the preservation of the spacial structure of images, CDAE is defined as

(4) $$\begin{array}{cc} E_{w_{e}}\left(x\right) & =a(w_{e}^{T}*x)\equiv h \end{array}$$

(5) $$\begin{array}{cc} D_{w_{d}}\left(h\right) & =a(w_{d}^{T}*h) \end{array}$$

where now x and h are matrices or tensors, and “*” is the convolution operator.

**Figure 5:**
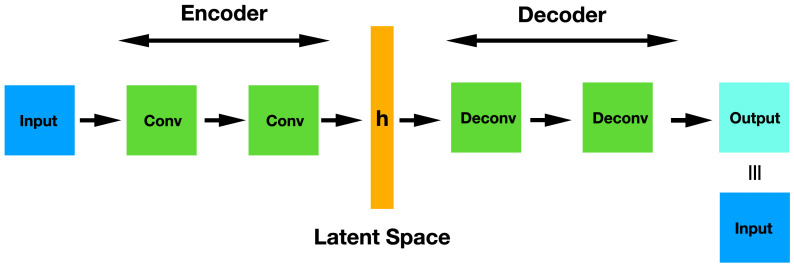
CAE architecture showing the convolutional encoder layers, the latent space and the deconvolutional decoder layers.

The architecture of a simple CAE is represented in Figure 5. This network can be pre-trained and used for classification tasks just in the same way as a Stacked Denoising Autoencoder does. Basically, once the complete CAE has been trained in an unsupervised manner, you throw away the decoder part and use only the encoder. Thus, these original weights are frozen and the hidden representation h is used as input in a MLP that carries out the final classification task.

### 2.4. Ensemble with *K*-NN Voting

A voting ensemble is an ensemble machine learning model that combines the predictions from multiple other models. It is generally used to improve final performance of the entire model rather than just using individual ones. It can be used for classification or regression problems, combining the labels or averaging predictions, depending on the case. There are two different approaches to the majority vote prediction for classification: hard and soft voting. In hard voting the class is predicted with the largest sum of votes from models whereas in soft voting it is predicted with the largest summed probability from models.

Here we propose a novel soft voting method that combines the probability of class membership obtained by the three different image processing models mentioned above. To do that, once they are trained, a weighted average is used to get the final class probability. As the output layer of each classifier is softmax and they are trained to minimize an entropic loss, we can consider each output component as an estimator of the posterior class probability. As it was previously commented, vectors **h** in latent spaces are classified by means of an MLP network. The output y of the MLP is a *C*-dimensional vector, being *C* the number of classes of the problem. In this way, the prediction of the ensemble for the *i*-th input sample is given by

(6) $$y^{i}=w_{cnn}^{i}⊙y_{cnn}^{i}+w_{caps}^{i}⊙y_{caps}^{i}+w_{cae}^{i}⊙y_{cae}^{i}$$

where “⊙” symbol represents the Hadamard product and $y_{cnn}^{i}$, $y_{caps}^{i}$ and $y_{cae}^{i}$ are the class estimations of the convolutional neural network, capsule network and convolutional autoencoder, respectively. $w_{cnn}^{i},w_{caps}^{i}\;\mathrm{and}\;w_{cae}^{i}$ are *C*-dimensional weighting vectors that indicate how reliable the class estimates of each network for sample *i*-th is. To determine them, the *K* Nearest Neighbors (*K*-NN) algorithm is used. Thus, given a particular model and the *i*-th sample in the training set, the *K* nearest neighbors to this sample in the latent space are found. Then, the weighting values are obtained from the known labels of nearest samples dividing the numbers of samples of each class by *K*. Thus, a large value of the *j*-th component of $w^{i}$ means that, in the neighborhood of the *i*-th sample in latent space, there are a large number of samples belonging to class *j*-th class. Note that, both $y_{model}$ and **w** vectors can be considered as probabilities because the sum of theirs components is one, so vector y can also be easily normalized in such a way that its components represent probabilities too.

The final ensemble output will be the class corresponding to the maximum component in $y^{i}$.

## 3. Results and Discussion

In this section, the performance of the considered machine learning models will be analyzed to validate the proposal. To do this, two different retinal images datasets will be used: one to compare the proposed algorithms with the state-of-the-art results obtained over a common dataset, and another one to apply these methods over a practical dataset. During training, images were normalized between 0 and 1 in both cases, and then a 10-fold Cross Validation (CV) was carried out. Every training was maintained during 200 epochs applying the early stopping process using a batch size of 16 images. In order to maximize the model performance with respect to *K* in the ensemble *K*-NN voting scheme, a search over the values K={3,5,7,10} is performed.

### 3.1. Fundus Images Datasets

Here the aforementioned datasets will be presented in more detail. On the one hand, a public, large and heterogeneous dataset (JOINT dataset) will first allow us to optimize the networks over a binary classification problem with enough data, avoiding data augmentation techniques. On the other hand, the second considered dataset (PAPILA dataset) is composed by a reduced number of images acquired in clinical practice conditions, since it is part of two research projects currently in progress. Due to be a small dataset a specific data augmentation technique will be needed to improve classification results.

#### 3.1.1. JOINT Dataset

In order to check the ability of the proposed network to distinguish generic glaucomatous retinal images from normal ones, we use part of the aggregated dataset proposed by Díaz-Pinto et al. [20], that contains Regions of Interest (ROI) extracted from different heterogeneous public fundus datasets in which the images are divided into Normal and Glaucoma categories. Authors carried out a process of cropping all the fundus images in a similar way and conditions, discarding images with unusable bright, resolution or visibility. Nevertheless, as showed in Table 1, in this work only the 2357 images that were originally labelled are taken and exploited from 86,926 total images enclosed in Díaz-Pinto dataset.

The JOINT dataset is completed with more ROIs of fundus images from other four different datasets which are described in Table 1. Because of they have nonuniform dimensions, they were resized to achieve a common and practical spatial resolution of 200 × 200 pixel, using bicubic interpolation.

#### 3.1.2. PAPILA Dataset

Once the validity of the proposed networks is proved over the JOINT set, the same models will be applied over a novel dataset called PAPILA [26]. This is a dataset arising from two research projects that are being developed jointly with Hospital Reina Sofía, Murcia, Spain.

Nowadays, this dataset is composed of fundus images of both eyes of 244 patients, which makes a total of 488 images. The original resolution of the retinal images was 2576 × 1934 pixels, and they were taken by the TOPCON TRC-NW400 retinograph with a 45-degree opening angle, in a frontal and centered view. Every retinal image was annotated by an ophthalmologist expert to make possible the extraction of a ROI that includes the relevant information for glaucoma diagnosis. That ROI comprises the optic disc and the optic nerve excavation area. Besides, every fundus image was separately diagnosed by an expert ophthalmologist within 3 categories: 333 healthy (class 0), 87 confirmed glaucoma disease (class 1), and 68 suspicions of suffering glaucoma (class 2). This dataset is currently under review and pending of publication.

Due to the limited number of images, in the first step, an offline data augmentation process was applied to increase the dataset size by a factor of ten. From every full fundus image, ten croppings were made with different pairs of offset/rotation values, to achieve realistic and complete ROIs, avoiding false edge artifacts that arise when the augmentation operation was made by displacing-and-padding, instead of masking the source image. The random offset values were uniformly generated in the range of ±20 pixels, and the random rotations in the range of $\pm 15^{\circ }$ in the same way. Additionally, the color of augmented ROIs was subjected to a slight and subsequent standardization procedure, in which the final illumination and contrast of every ROI were adjusted to be in a normal distribution whose mean and variance were those of the original images.

### 3.2. Networks Architecture and Training

#### 3.2.1. CNN

According to the problem complexity, a basic CNN architecture proved to be adequate in this work. In particular, a CNN composed of two convolutional layers with 32 and 64 filters respectively, both with 3 × 3 convolutional kernels, and a max pooling layer between them was selected. The pooling layer downsamples the data by taking the maximum value over a 2 × 2 window. Finally, a MLP with 1024 hidden and 3 output neurons were used to achieve the classification. The ReLU activation function was used in all the layers, except for the output of the MLP, where softmax is needed with the aim of classifying the fundus images. The model was trained minimizing the Cross Entropy loss function with Adam optimizer.

#### 3.2.2. CapsNet

A convolutional layer with 8 filters and ReLU activations was chosen for the first part of the CapsNet. This section connects to a PrimaryCaps layer of 8 filters and 1 channel, followed by a DigitCaps layer of 16 capsules. In this work, a fourth step is carried out using a MLP to reconstruct the input data from the DigitCaps output. This has demonstrated to help to get better final classification results. For this task, a MLP with a hidden layer of 1024 neurons is used. Therefore, the entire network is trained to solve two tasks simultaneously (the classification and the reconstruction) by using the Adam optimizer and combining the minimization of the margin loss error function (classification) and the MSE (reconstruction) through a convex combination.

#### 3.2.3. CDAE

The CDAE consists of an encoder with two sequential convolutional layers with 32 and 64 filters respectively, and an opposite symmetric reconstruction decoder. The whole network was trained contaminating the input images using AWGN with mean 0.5 and standard deviation 0.5, and then minimizing the MSE error function by means of the Adam optimizer. In a second training stage, the encoder weights were frozen, and the output **h** was connected to a MLP with a hidden layer of 1024 neurons and an output layer with softmax activations. This joint network was then trained with Adam optimizer to minimize the Cross Entropy loss function for classification purposes.

### 3.3. Experiments

As previously stated, the main goal of this work is to demonstrate that deep learning and ensemble learning methods are able to improve state-of-the-art results in Glaucoma image classification. Thus, in order to show the performance of the models for each dataset, we will show Receiver Operating Characteristic (ROC) curves and Area Under the Curve (AUC) measurements reached by every deep network. It is widely known that these metrics are suitable in problems like these. ROC is a probability curve and AUC represents the degree or measure of data separability. These show how much each model is capable of distinguishing between classes in a binary scenario. The higher the AUC, the better the model is at predicting 0 s as 0 s and 1 s as 1 s. By analogy, the higher the AUC, the better the model is at distinguishing between patients with the disease and those that are healthy. In addition, to fully understand the behavior of the corresponding models, sensitivity and specificity metrics will be presented and analyzed.

#### 3.3.1. JOINT Dataset

Table 2 collects the performance of all models with the JOINT dataset in terms of AUC, sensitivity and specificity, obtained for the best decision threshold according to the G-mean metric [27], which is intended to balance sensibility and specificity. The results are presented in mean and standard deviation after a 10-fold CV, and the best result for each metric and each model is shown in bold type. According to this criteria, it can be seen how the ensemble method produces the best overall results. It gets the highest average value of AUC of 0.98 (for K=10), and the best sensibility and specificity results equal to 0.94 and 0.97, respectively.

**Figure 6:**
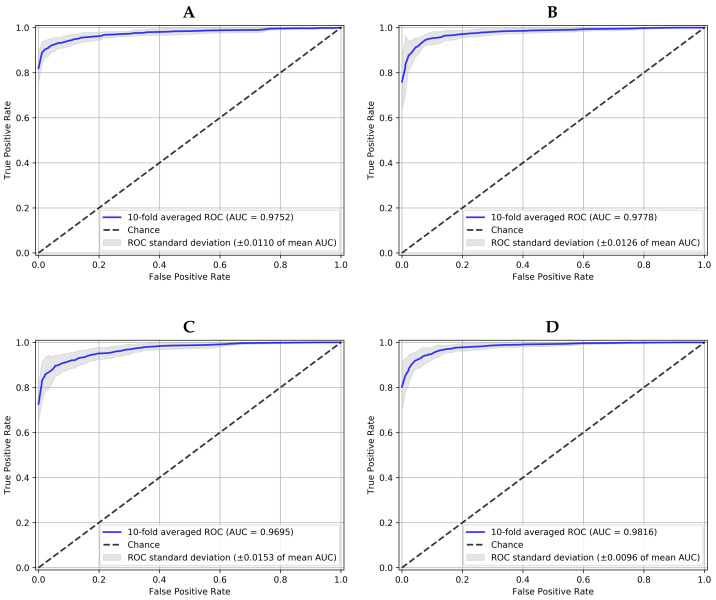
10-fold averaged ROC for positive class (Glaucoma) and each model working over JOINT dataset. (**A**) is the Convolutional Neural Network, (**B**) the Capsule Network, (**C**) the Convolutional Denoising Autoencoder and (**D**) the Ensemble with 10-NN Voting.

To graphically observe these results, Figure 6 represents the averaged ROC curves of the 10-fold CV for each network. As it has been mentioned above, the proposed 10-NN voting ensemble was the best one in terms of averaged AUC (>0.98) and averaged standard deviation (<0.01). This result outperforms the AUC of 0.9605 achieved in the reference work [25], with the same dataset. To the best of our knowledge, no other works evaluate the same dataset. In that work, an average sensitivity of 0.9346 and an average specificity of 0.8580 were also obtained without any decision threshold optimization. That is the reason why according to Table 2 a noticeably better sensitivity and specificity were attained here.

#### 3.3.2. PAPILA Dataset

The performance of all models with the PAPILA dataset are now shown in Table 3, in terms of averaged AUC, sensitivity and specificity between classes. As in the case of the JOINT dataset, the best values were obtained by the Ensemble method for all metrics. Furthermore, taking the “glaucoma” class as a reference and recomputing all the metrics with respect to it, in a One-vs-Rest approach, the proposed model still keeps the best sensitivity for detecting the disease with a value of 0.87 ± 0.04; by contrast, the CNN, CapsNet and CDAE models obtained 0.82 ± 0.10, 0.84 ± 0.12, and 0.86 ± 0.12, respectively.

From the results obtained with CapsNet both in PAPILA and JOINT, it can be concluded that equivalence does not seem to be a key point to improve the detection of glaucoma in retinographies with respect to other models. A plausible explanation for this result can be found in the almost circular symmetry of the objects present in the fundus images.

**Figure 7:**
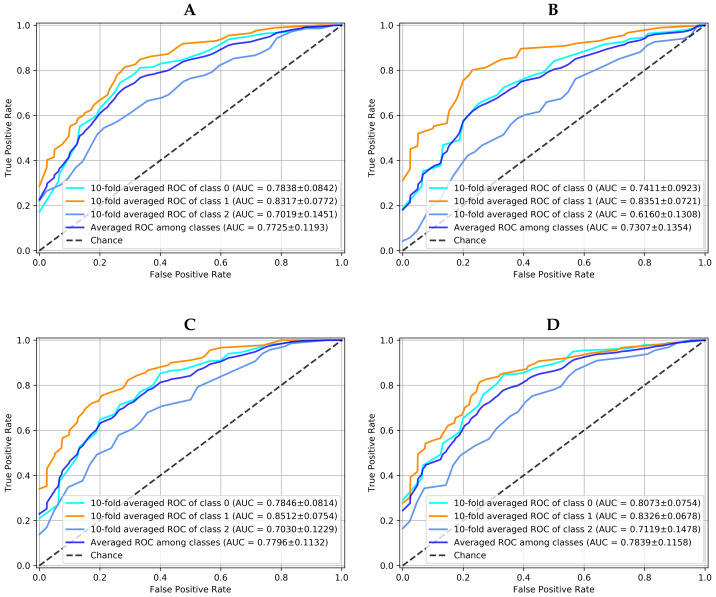
10-fold averaged ROC for each class and each model working over PAPILA dataset. Averaged ROC among classes for each model is also showed. (**A**) is the Convolutional Neural Network, (**B**) the Capsule Network, (**C**) the Convolutional Denoising Autoencoder and (**D**) the Ensemble with 10-NN Voting.

The graphical results for this practical dataset using the data augmentation previously described are presented in Figure 7. This dataset represents a more challenging situation, taking into account that it is a classification problem with three classes. Besides, one of them (suspicious) supposes an undetermined clinical diagnostic that tends to confuse the classification decision. Figure 7 shows the 10-fold averaged ROC curve of each class and averaged ROC among classes for each method. It can be seen that, again, the proposed ensemble with *K*-NN voting method (K=10) is the best option to solve this problem since it is the one with the highest AUC for the averaged ROC among classes, with a ROC standard deviation close and similar in all models.

## 4. Conclusions

In this article, a novel soft voting method that combines the probability of class membership obtained by several deep learning models has been presented to detect the glaucoma disease. Looking for diversity, three different models have been chosen to form the final ensemble proposal: CNN, CapsNet and CDAE. By an appropriate combination of the estimations of the class probabilities provided by the three models, using the *K*-NN algorithm in the latent space of each model, the diagnosis of glaucoma can be improved with respect to those provided by the individual models. This conclusion has been validated by analyzing the results obtained on two datasets of retinal images: JOINT and PAPILA. According to the results, the equivariance property of the CapsNet seems not to be an important issue in the glaucoma problem. Finally, the proposed model allows to adjust the sensitivity-specificity results by searching for the appropriate values of decision threshold in the ROC curves and *K* in the *K*-NN method.

## Figures and Tables

**Table 1:** Contents of JOINT dataset, that was built from fundus image ROIs taken from the proposal of Díaz-Pinto et al. [20]. The original source and number of retinal ROIs are also showed.

Source Dataset	Glaucoma	Normal	Total
Drishti-GS1 [21]	70	31	101
RIM-ONE [22]	194	261	455
sjchoi86-HRF [23]	101	300	401
HRF [24]	27	18	45
ACRIMA [25]	396	309	705
JOINT dataset	788	919	1707

**Table 2:** Models performance (mean ± standard deviation over a 10-fold CV) obtained with the JOINT dataset in the Glaucoma class. For each model, the best result is shown in bold.

	AUC	Sensitivity	Specificity
CNN	0.97 ± 0.01	0.92 ± 0.02	0.96 ± 0.02
CapsNet	0.97 ± 0.01	0.93 ± 0.02	0.95 ± 0.02
CDAE	0.96 ± 0.01	0.89 ± 0.04	0.96 ± 0.02
Ensemble (K=10)	**0.98 ± 0.01**	**0.94 ± 0.02**	**0.97 ± 0.02**

**Table 3:** Models averaged performance (mean ± standard deviation over a 10-fold CV) obtained with the PAPILA dataset. For each model, the best result is shown in bold.

	AUC	Sensitivity	Specificity
CNN	0.77 ± 0.11	0.77 ± 0.12	0.73 ± 0.10
CapsNet	0.73 ± 0.13	0.74 ± 0.16	**0.74 ± 0.14**
CDAE	0.77 ± 0.11	0.79 ± 0.15	0.73 ± 0.12
Ensemble (K=10)	**0.78 ± 0.11**	**0.81 ± 0.15**	**0.74 ± 0.09**

## Data Availability

The JOINT dataset can be obtained from the original proposal [20], by selecting only the labeled images from this source. A paper describing the PAPILA dataset is currently accepted [26] and will be publicly available shortly.

## Abbreviations

The following abbreviations are used in this manuscript:

CNN	convolutional neural network
CapsNet	Capsule Network
CDR	Cup-to-Disk Ratio
ML	Machine Learning
DL	Deep Learning
DNN	Deep Neural Network
SDAE	Stacked Denoising Autoencoder
MLP	Multilayer Perceptrons
AE	Autoencoder
CAE	Convolutional Autoencoder
MSE	Mean Squared Errors
CDAE	Convolutional Denoising Autoencoder
ReLU	Rectified Linear Unit
ROI	Region of Interest
AWGN	Additive White Gaussian Noise
AUC	Area Under the Curve
ROC	Receiver Operating Characteristic
CV	Cross Validation
